# Decremental responses in patients with motor neuron disease

**DOI:** 10.1002/brb3.846

**Published:** 2017-09-26

**Authors:** Mohammed H. Alanazy, Janka Hegedus, Chris White, Lawrence Korngut

**Affiliations:** ^1^ Department of Internal Medicine King Saud University Medical City and College of Medicine King Saud University Riyadh Saudi Arabia; ^2^ Division of Neuromuscular Department of Clinical Neurosciences University of Calgary Calgary Alberta Canada

**Keywords:** amyotrophic lateral sclerosis, motor neuron disease, neuromuscular junction, progressive muscular atrophy, repetitive nerve stimulation

## Abstract

**Objective:**

Involvement of the neuromuscular junction (NMJ) in amyotrophic lateral sclerosis (ALS) has been reported and is increasingly recognized as an important pathophysiological aspect. The relationship between decrement and clinical measures for possible application as a biomarker has not been comprehensively explored.

**Methods:**

We performed routine repetitive nerve stimulation (RNS) of three nerves on patients with ALS. We captured measures of muscle strength, grip strength, fatigability, and calculated slow vital capacity (SVC) rates of change assessing for associations.

**Results:**

In 42 subjects, 210 muscles were studied. Negative correlation was found between the percentage of decrement and compound muscle action potential (CMAP) amplitude. Approximately half of the patients with hand weakness did not have decrement. There was no significant correlation between decrement and handgrip fatigue, SVC < 80% predicted, or more rapid worsening of SVC over time.

**Conclusions:**

Abnormal decremental responses are well described in ALS. We report that the degree of decremental response does not correlate with the degree of weakness. Abnormal decrement is only rarely present in nerve–muscle pairs with normal motor power. Our findings did not support a correlation between abnormal decrement and clinical measures suggesting that RNS may not be useful as a biomarker to monitor ALS progression.

## 1 INTRODUCTION

Amyotrophic lateral sclerosis (ALS) is a motor neuron disease (MND) that results in rapidly progressive quadriparesis, muscle atrophy, and respiratory failure, typically leading to death within 3–5 years. The incidence of ALS in Canada is reported to be 2.4 per 100,000 (Wolfson, Kilborn, Oskoui, & Genge, 2009).

Clinical and electrophysiological correlates of muscle fatigability suggesting an element of neuromuscular junction (NMJ) transmission dysfunction have been described in patients with ALS over the past 50 years (Mulder, Lambert, & Eaton, 1959). Decremental responses in ALS have different characteristics from classic presynaptic (Lambert–Eaton myasthenic syndrome) and postsynaptic (myasthenia gravis) disorders of NMJ transmission (Killian, Wilfong, Burnett, Appel, & Boland, 1994). Killian et al. (1994) concluded that low baseline compound muscle action potential (CMAP) amplitudes with decrement may suggest a presynaptic transmission deficit. Stålberg (1982) hypothesized that reinnervation of motor endplates with reduced acetylcholine stores could cause decremental responses. As such, decremental responses should be expected in the vast majority of cases when followed longitudinally, but that was not observed in a case series of 30 cases over a mean of 18 months, suggesting that decrement may not be solely explained by sprouting and other variables influencing acetylcholine release may contribute to the decremental response (Killian et al., 1994). Molecular and pathological studies are increasingly demonstrating an important role of the NMJ early in the pathophysiology of ALS involving cell adhesion molecules that normally stabilize the NMJ mediated by proteins like adducin (Krieger, Wang, Yoo, & Harden, 2016).

In this study, we investigated patterns of decremental responses in patients with MND (ALS and progressive muscular atrophy [PMA]) and conducted comparisons with a detailed set of clinical measures including Medical Research Council (MRC) scale, grip strength, handgrip fatigue time, ALS Functional Rating Scale‐Revised (ALSFRS‐R), and slow vital capacity (SVC), and examined the relationship between decremental responses in trapezius and SVC. While abnormal decremental responses are well described in ALS, the aim of this study was to explore their presence and relationship with clinical measures for possible use as a biomarker for clinical findings in ALS cohort studies and clinical trials.

## 2 METHODS

### 2.1 Participants

The study was approved by the University of Calgary Conjoint Health Research Ethics Board (CHREB). Subjects were identified during routine clinic visits to the Calgary ALS and MND clinic (Calgary, Alberta). All patients provided signed informed consent prior to initiation of any study procedures. Inclusion criteria were as follows: (1) definite, probable, clinically probable laboratory‐supported, or clinically possible ALS according to the revised El‐Escorial diagnostic criteria (Brooks, Miller, Swash, & El Munsat, 2000); (2) PMA (progressive muscle weakness secondary to anterior horn cell involvement confirmed by clinical and electrophysiological examination, and no clinical evidence of upper motor neuron signs); and (3) age above 18 years. We excluded patients with diagnosed NMJ disorders, myopathies, neuropathies, primary lateral sclerosis, and spinal bulbar muscular atrophy. All studies were performed on a Cadwell Sierra Wave EMG machine (Cadwell Laboratories Inc., Kennewick, WA).

### 2.2 Repetitive nerve stimulation

Repetitive nerve stimulation (RNS) was performed by an experienced EMG technologist blinded to the results of the other outcome measures using surface electrodes with belly tendon technique for CMAP recording (Preston & Shapiro, 2013). Skin temperature was maintained >32°C over abductor pollicis brevis (APB) and abductor digiti minimi (ADM). RNS was performed over five muscles (bilateral APB and ADM, and unilateral trapezius). Muscles with CMAP amplitude <1.0 millivolt (mV) were excluded from RNS study. A single stimulation (0.2 millisecond duration) was recurrently administered with increasing current to establish supramaximal stimulation followed by a train of six supramaximal stimulations administered at 3 Hz to each nerve at baseline, followed by 1 min of maximal isometric exercise, followed by a train of RNS provided immediately and at 1, 2, 3, and 4 min postexercise. CMAP decrement was calculated as the maximum difference between the first and fourth CMAP negative peak amplitude and expressed as a percentage. We considered a significant decrement to be ≥10.0%. When decrement exceeded 10.0%, RNS was repeated after 10 s of isometric exercise and evaluated for repair (improvement of the absolute size of decrement by ≥2%).

A decremental response of 10% has long been established as a clinical cut point to indicate an abnormal result and in our clinical experience decremental responses between 5% and 7.5% are not unusual in subjects without NMJ transmission dysfunction. In patients with MND, the CMAP amplitude is usually small; therefore, a small drop in its amplitude, which might be due to the inherent variability of the test, could translate into a positive decrement. For example, a drop of 0.05 mV would be considered significant (5%) in a muscle with a CMAP amplitude of 1.0 mV. RNS is technically challenging and is more difficult in those muscles with small CMAP amplitudes. In our opinion, the cut point of 10% reduces the association between ALS and abnormal decrement, perhaps resulting in somewhat of an underestimate, but a more trustworthy result.

In addition to patient demographics, we captured the following data on the RNS study visit day: (1) site of disease onset (limb or bulbar) and disease duration; (2) percent‐predicted SVC at baseline and follow‐up, with a normal range considered to be between 80% and 120% (Barreiro & Perillo, 2004); (3) fatigability measured using a JAMAR^®^ hand grip dynamometer (Sammons Preston Roylan, Bolingbrook, IL) (Roberts et al., 2011), and expressed as the time in seconds to drop from 30% to 20% of maximal hand grip strength; (4) ALSFRS‐R score (Cedarbaum et al., 1999); and (5) MRC scale for APB, ADM, and first dorsal interosseous (FDI).

### 2.3 Statistical analysis

The proportion of patients with significant decrement in any muscle and the proportion of muscles with significant decrement with or without repair after brief exercise were calculated. The Pearson's correlation test was performed to examine for correlation between parametric variables. An independent *t* test and ANOVA with post hoc Tukey were performed to compare means of parametric variables. Results were reported as group means ± standard deviation (*M* ± *SD*). A Mann–Whitney *U* test was performed to compare the differences between the ordinal MRC scale data of APB and ADM. A *p* < .05 was considered statistically significant. In our laboratory, lower limit normal (LLN) of CMAP amplitude values for APB and ADM were 4.7 and 7.8 mV, respectively. We calculated odds ratios and 95% confidence intervals (CIs) to assess the association between the presence of significant decrement (exposure variable) in the trapezius muscle and SVC <80% (disease variable). Odds ratio *p*‐values were calculated and were considered significant if they were below 0.05 and if their 95% CI did not cross the null value of 1.0. Calculations were done with SPSS (version 20 for Windows) software.

## 3 RESULTS

We studied a total of 210 nerve–muscle pairs (84 APB, 84 ADM, and 42 trapezius) in 42 patients (28 males and 14 females). Six patients met criteria for PMA and 36 for ALS. Alternative diagnoses had been ruled out through appropriate clinical assessment, EMG studies, blood work, and neuroimaging. There was no significant difference between patients with limb (30 patients) and bulbar (12 patients) onset MND in the means of decremental responses, ALSFRS‐R score, trapezius decrement, SVC, grip strength, and handgrip fatigue time (Table 1). After excluding muscles with CMAP < 1 mV, RNS was performed on 165 nerve–muscle pairs (58 APB, 66 ADM, and 41 trapezius). Abnormal decrement was present in 29.3% of APB, 9.1% of ADM, and 24.4% of trapezius muscles (Table 2).

**Table 1:** Demographics and comparison between limb‐ and bulbar‐onset MND

	All subjects (*n* = 42) (mean ± *SD*)	Limb onset (*n* = 30) (mean ± *SD*)	Bulbar onset (*n* = 12) (mean ± *SD*)	*p* value Limb vs. bulbar onset
Age, years	61.1 ± 9.8	59.9 ± 9.8	64.0 ± 9.7	.23
Sex, (*n* = male, female)	28, 14	21, 9	7, 5	NA
Disease duration, months	32.8 ± 28.1	38.8 ± 29.7	18.3 ± 17.4	.03
CMAP amplitude	4.2 ± 2.9	3.5 ± 2.9	5.8 ± 2.1	<.0001
RNS APB decrement (%)	8.9 ± 6.6	9.4 ± 7.2	8.2 ± 5.8	.50
RNS ADM decrement (%)	5.1 ± 3.6	5.7 ± 3.6	4.0 ± 3.4	.06
RNS trapezius decrement %	8.5 ± 6.3	9.1 ± 5.7	7.0 ± 7.7	.35
SVC%	89.1 ± 24.2	87.2 ± 25.5	93.6 ± 21.0	.45
Grip strength	42.4 ± 34.9	37.5 ± 37.6	54.5 ± 23.7	.054
Handgrip time to fatigue, seconds	60.3 ± 45.8	52.8 ± 43.6	73.2 ± 47.7	.10
ALSFRS‐R	37.2 ± 6.0	36.4 ± 6.6	39.1 ± 3.7	.20

ADM, abductor digiti minimi; ALSFRS‐R, ALS functional rating scale‐revised; APB, abductor pollicis brevis; CMAP, compound muscle action potential; NA, not applicable; SVC%, slow vital capacity percentage of predicted value; RNS, repetitive nerve stimulation.

**Table 2:** Decremental responses and CMAP amplitude per muscle and MRC scale

	Median MRC score	CMAP (mV) Mean ± *SD*	Decrement^a^ Mean % ± *SD*	Muscles with decrement ≥ 10%, *n* (%)	Decrement repair postbrief exercise, *n* (%)
APB	4.0	3.7 ± 3.1	8.9 ± 6.6	17/58 (29.3)	15/17 (88.2)
ADM	4.0	4.6 ± 3.1	5.1 ± 3.6	6/66 (9.1)	2/6 (33.3)
Trapezius	NA	4.3 ± 1.7	8.5 ± 6.3	10/41 (24.4)	6/10 (60.0)
MRC5	NA	7.1 ± 1.4	4.2 ± 2.6	1/26 (3.8)	1/1 (100)
MRC4	NA	5.8 ± 2.2	6.6 ± 5.4	14/78 (17.9)	11/14 (78.6)
MRC ≤ 3	NA	0.9 ± 1.2	11.1 ± 6.7	8/20 (40.0)	5/8 (71.4)

ADM, abductor digiti minimi; APB, abductor pollicis brevis; MRC, Medical Research Council.

a From RNS study, 44 muscles with CMAP <1 mV were excluded.

**Figure 1:**
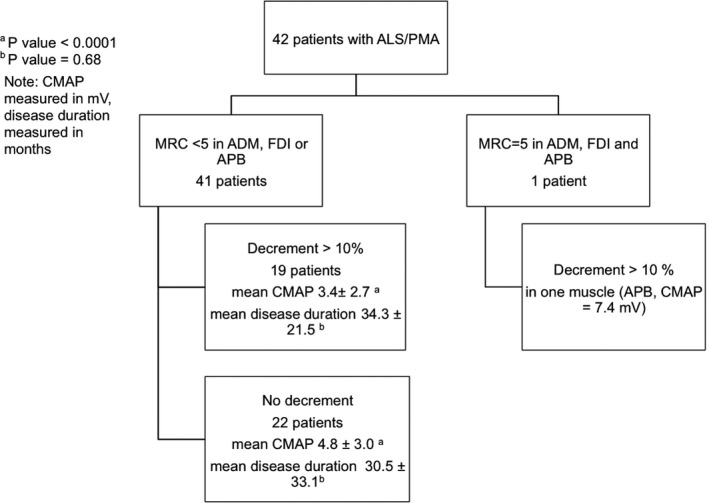
Study participants. Flow diagram of the study participants grouped by MRC scale and decrement

A significant decrement in at least one muscle was observed in 20/42 (47.6%) patients. Forty‐one patients (97.6%) had weakness in APB, ADM, and/or FDI, but only 19 (46.3%) of them had abnormal decrement in one or more muscles (i.e., weakness and decrement, W + D), and only one patient had decrement in a strong APB muscle (Figure 1). Twenty‐two of the 41 (53.7%) patients had weakness with no significant decrement (W − D). W + D patients had significantly lower mean CMAP amplitude (3.4 ± 2.7) than the W − D patients (4.8 ± 3.0) (*p* < .0001). There was no significant difference in disease duration between the two groups (*p* = .68).

Clinical and electrophysiological findings by muscle group were analyzed and there were no significant differences in CMAP amplitudes or MRC scale (Table 2). Mean decremental responses were 8.9 ± 6.6% for APB, 5.1 ± 3.6% for ADM, and 8.5 ± 6.3% for trapezius (Table 2). Analysis with ANOVA demonstrated significantly higher mean decrement in APB than ADM (*p* = .001), and no difference between mean decrement of APB and trapezius (*p* = .94), whereas trapezius decrement was significantly higher than ADM (*p* = .006). A sample of the original data is provided in Table S1.

ANOVA analysis revealed a significantly higher decrement in the APB and ADM with MRC ≤ 3 (11.1 ± 6.7%) than those with MRC 4 (6.6 ± 5.4%; *p* = .004) or MRC 5 (4.2 ± 2.6%; *p* < .0001). Decrement in APB and ADM with MRC 4 was greater than those with MRC 5, but the difference was not statistically significant (*p* = .09) (Table 2).

We investigated the relationship between trapezius decremental responses and SVC. Twelve patients had SVC <80% of predicted value at the time of the study, five of them had significant decrement in the trapezius. The odds ratio of having an SVC <80% among patients with abnormal trapezius decrement was 4.17 (95% CI: 0.91–19.2; *p* = .07). The mean percent‐predicted SVC was significantly lower in the group with significant trapezius decrement (77.9 ± 23.8%) than the group with no decrement (94.4 ± 21.5%; *p* = .046). A follow‐up SVC was obtained for 30 patients, 5 of them had decrement and 25 had no decrement in trapezius muscle at baseline, at 4.5 ± 2.1 and 5.4 ± 1.6 months, respectively (*p* = .29).

There was no significant difference in the rate of change of percent‐predicted SVC/month between the group with (−0.5 ± 1.9%) and without (−1.3 ± 2.3%) trapezius decrement at baseline (*p* = .49). There was also no significant difference in the rate of change of percent‐predicted SVC/month between a group of 14 patients with significant decrement in any muscle (−0.6 ± 1.7%) and a group of 17 patients with no decrement at baseline (−1.5 ± 2.5) (*p* = .25).

Correlation analysis revealed a negative correlation between the degree of decrement in APB, ADM, and trapezius and their CMAP amplitude (*r* = −.53, *p* < .0001), and between the degree of the decrement in APB and ADM and grip strength (*r* = −.46, *p* = .001). There was a positive correlation between grip strength and handgrip time to fatigue (*r* = .51, *p* < .0001). No significant correlations were observed between the size of decrement in APB and ADM and handgrip time to fatigue (*r* = −.17, *p* = .25). No significant correlations were observed between the size of decrement in APB, ADM, and trapezius and age (*r* = .01, *p* = .95), disease duration (*r* = .06, *p* = .76), ALSFRS‐R scores (*r* = −.02, *p* = .92), or SVC (*r* = −.15, *p* = .46). Trapezius CMAP amplitude had a moderate correlation with SVC (*r* = .56, *p* < .001).

## 4 DISCUSSION

Abnormal decremental motor responses on RNS are not specific to myasthenia gravis as many studies have demonstrated neuromuscular transmission impairment in patients with ALS (Bernstein & Antel, 1981; Henderson, Baumann, Hutchinson, & McCombe, 2009; Henderson & Daube, 2004; Iwanami et al., 2011; Killian et al., 1994; Kim, Park, Kim, & Sunwoo, 2011; Mulder et al., 1959; Stålberg, 1982; Wang, De Pasqua, Gérard, & Delwaide, 2001; Yamashita et al., 2012). Abnormal decremental responses have been observed more frequently in proximal than distal muscles (Iwanami et al., 2011; Killian et al., 1994). In one study, greater decrement was observed in a group of rapidly progressive disease (6 patients) than slowly progressive disease (8 patients) (Bernstein & Antel, 1981). Other studies showed no apparent difference in survival between the patients with and without decrement (Henderson et al., 2009; Wang et al., 2001). Our finding of similar disease duration between the W + D and W − D patients is consistent with previous studies. One reason is that our cohort included mostly patients with slow progression (mean disease duration 32.8 ± 28.1 months, mean ALSFRS‐R 37.2 ± 6.0, and mean SVC 89.1 ± 24.2) due to the inherent selection bias of referral clinics. That said, we probably missed those patients who had a rapidly progressive disease and passed away before the time of our study or not referred to the ALS clinic for any reason.

Only about half of our patients who had weakness in the hand muscles were found to have significant decrement. This finding reinforces the fact that the MRC grading scale is not linear and a rather crude way to monitor muscle strength or quantify motor unit loss. The CMAP may be better than MRC scale in monitoring the progression of the disease as it reflects the number of muscle fibers that could be activated by the stimulated nerve. In this study, the size of decrement was inversely correlated with CMAP amplitude, and the W + D patients had significantly lower CMAP amplitude than the W − D patients. We speculate that decrement develops at a threshold when a significant number of motor units have undergone degeneration in the presence of immature collateral sprouting from adjacent motor units that are undergoing an active degeneration process. This results in a low safety factor and insecure neuromuscular transmission. This is supported by findings from other studies that showed a more frequent and greater size of decrement in atrophic muscles (Denys & Norris, 1979; Mulder et al., 1959) as well as in muscles with small CMAP (Henderson et al., 2009; Iwanami et al., 2011; Killian et al., 1994; Wang et al., 2001). In a previous study, the size of decrement predicted further drop in CMAP amplitude, indicating a possible role for immature reinnervation and reduced safety factor in the pathogenesis (Wang et al., 2001).

We investigated the association between trapezius decrement and percent‐predicted SVC, with a hypothesis of finding a strong association based on their shared innervation from the upper cervical segments (Svenberg Lind et al., 2015). The presence of significant decrement in the trapezius at baseline demonstrated a trend toward a correlation with low SVC (<80%) at the first visit, but did not predict more rapid rate of change in percent‐predicted SVC/month in comparison to the group with no decrement at baseline. In addition, we found a moderate positive correlation between trapezius CMAP amplitude and SVC at baseline. We thus suggest, with the caveat that our small sample size limited the precision of analysis, that the presence of a significant decrement in the trapezius may be associated with a low SVC, but is not predictive of more rapid subsequent decline in SVC. Likewise, the presence of significant decrement in any muscle (APB, ADM, trapezius) does not predict further drop in percent‐predicted SVC/month. Further studies are required to investigate the relation between trapezius CMAP amplitude and decrement with SVC.

## 5 CONCLUSIONS

The main aim of this study was to examine RNS decremental responses as a possible surrogate marker of clinical findings for use in ALS clinical trials. Although we replicated findings from previous studies, abnormal decremental responses were not associated with clinical measures to suggest promise in the monitoring of clinical status or disease progression in ALS. While abnormal decremental responses are well described in ALS, we report that the degree of decremental responses does not correlate with degree of weakness and that abnormal decrement is only rarely present in nerve–muscle pairs with normal motor power. As we did not investigate RNS in specific types of ALS, such as patients with a fast‐progressing disease or with a specific gene mutation, a biomarker role of RNS in these subtypes of ALS cannot be excluded based on this study.

## CONFLICT OF INTEREST

None.

## AUTHORS’ CONTRIBUTIONS

All authors conceived and participated in the design of the study. MA and JH collected the data. MA and LK carried out the analyses. JH, LK, and CW supported the analyses and interpretation of the study results. MA, JH, LK, and CW drafted the manuscript. MA and LK carried out the revision of the manuscript. All authors read and approved the final manuscript.
