# Supplementary material for: Decremental responses in patients with motor neuron disease
Source: Brain Behav. 2017 Sep 26;7(11):e00846. doi: 10.1002/brb3.846 (PMC5698864; doi:10.1002/brb3.846)
Supplement: Supplementary file 1 [file BRB3-7-e00846-s001.docx]

Supplementary Table. A sample of the original data of abductor pollicis brevis MRC scale and the corresponding CMAP amplitude and decrement.

| Patient | Muscle | MRC scale (0-5) | CMAP amplitude (mV) | RNS decrement % |
| --- | --- | --- | --- | --- |
| 1 | APB | 3 | 1.05 | 23.1 |
| 2 | APB | 1 | 0.15 | NA |
| 3 | APB | 1 | 0 | NA |
| 4 | APB | 4 | 6.62 | 9.3 |
| 5 | APB | 5 | 7.06 | 3.7 |
| 6 | APB | 5 | 8.44 | 0 |
| 7 | APB | 5 | 9.85 | 4.1 |
| 8 | APB | 3 | 1.43 | 8.6 |
| 9 | APB | 0 | 0 | NA |
| 10 | APB | 5 | 6.09 | 4.3 |
